# Multi-Scale Characterization of Lyotropic Liquid Crystals Using ^2^H and Diffusion MRI with Spatial Resolution in Three Dimensions

**DOI:** 10.1371/journal.pone.0098752

**Published:** 2014-06-06

**Authors:** Diana Bernin, Vanessa Koch, Magnus Nydén, Daniel Topgaard

**Affiliations:** 1 Applied Surface Chemistry, Chalmers University of Technology, Gothenburg, Sweden; 2 Swedish NMR Centre, University of Gothenburg, Gothenburg, Sweden; 3 Ian Wark Research Institute, University of South Australia, Adelaide, South Australia, Australia; 4 Division of Physical Chemistry, Department of Chemistry, Lund University, Lund, Sweden; Martin-Luther-Universität Halle-Wittenberg, Germany

## Abstract

The ability of lyotropic liquid crystals to form intricate structures on a range of length scales can be utilized for the synthesis of structurally complex inorganic materials, as well as in devices for controlled drug delivery. Here we employ magnetic resonance imaging (MRI) for non-invasive characterization of nano-, micro-, and millimeter scale structures in liquid crystals. The structure is mirrored in the translational and rotational motion of the water, which we assess by measuring spatially resolved self-diffusion tensors and 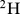 spectra. Our approach differs from previous works in that the MRI parameters are mapped with spatial resolution in all three dimensions, thus allowing for detailed studies of liquid crystals with complex millimeter-scale morphologies that are stable on the measurement time-scale of 10 hours. The 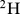 data conveys information on the nanometer-scale structure of the liquid crystalline phase, while the combination of diffusion and 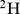 data permits an estimate of the orientational distribution of micrometer-scale anisotropic domains. We study lamellar phases consisting of the nonionic surfactant C_10_E_3_ in 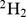
O, and follow their structural equilibration after a temperature jump and the cessation of shear. Our experimental approach may be useful for detailed characterization of liquid crystalline materials with structures on multiple length scales, as well as for studying the mechanisms of phase transitions.

## Introduction

Amphiphilic molecules such as surfactants and lipids spontaneously form a range of liquid crystalline phases when mixed with water [1], [2]. While the nanometer-scale structure is dictated by the temperature and the local chemical composition, the morphology on larger length scales is highly tunable through, e.g., the thermal history [3], the presence of magnetic fields [4]–[7], or the application of shear [8]–[14]. Micrometer-scale structures such as multi-lamellar vesicles (MLVs) are of interest as microreactors [15] and for drug-delivery applications [16], while the millimeter-scale organization is relevant when the liquid crystalline phase is used as a template for inorganic materials [17], [18]. After removing the sources of the perturbations, the formed superstructures are not at true thermodynamic equilibrium, but they may nevertheless be metastable for extended periods of time and useful for practical applications.

The interface between the water and the hydrophobic core of the surfactant aggregates imparts anisotropy to the rotational and translational motion of the water molecules. Although the orientational ordering of the water is minuscule, it can be detected through the exquisitely sensitive 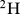 quadrupolar interaction using nuclear magnetic resonance (NMR) spectroscopy [19]. Not only can 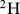 NMR be used for distinguishing between cubic, hexagonal, and lamellar liquid crystalline phases [20], [21], but also for determining the degree of orientational order [4]–[7], as well as the size [22]–[26] of the anisotropic microcrystallites. The features of the 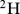 spectrum are sensitive to the orientation of the liquid crystalline phase with respect to the direction of the applied external magnetic field. For axially symmetric phases, e.g., hexagonal and lamellar, the angle between the magnetic field and the main symmetry axis of the phase determines the observed 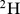 spectrum. Conversely, there is no information about the orientation within the plane perpendicular to the magnetic field, thus making it difficult to pinpoint the exact microcrystallite orientation in 3D space.

The translational diffusion of water is conveniently monitored with pulsed-gradient spin-echo (PGSE) NMR [27]–[29], in which the ^1^H NMR signal is encoded for molecular displacements using magnetic field gradients. Just as for 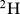 spectroscopy, the degree and length scale of the orientational ordering of the anisotropic microcrystallites can be determined using PGSE methods [30]–[33]. Modern NMR spectrometers usually have the capability of generating field gradients in three orthogonal directions, thus making it possible to determine the full diffusion tensor from which the preferred direction of microcrystallite orientation can be estimated [27], [34], [35].

Using magnetic resonance imaging (MRI) methods, both 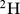 spectroscopy [36] and diffusion experiments [37], [38] can be performed in a spatially resolved manner. In the context of surfactant science, the spatial resolution has often been limited to a single dimension [39]–[41], with two examples of two-dimensional mapping of diffusion tensors [42], [43]. Combined one-dimensional mapping of 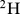 spectra and diffusion coefficients has been used to investigate transitions between various lamellar phase morphologies induced by the application of shear and temperature cycling [26], [40].

In this work, we investigate the benefit of full three-dimensional mapping of diffusion tensors and 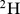 spectra for characterizing hierarchically organized liquid crystalline phases with intricate structures on the millimeter scale. As a model system we use the non-ionic surfactant triethylene glycol monodecyl ether (C_10_E_3_) and deuterated water (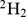
O), which has often been applied in studies of lamellar phase morphologies [13], [23], [24], [26], [33], [40], [43], [44]. Structures on a wide range of length scales are assessed by correlating the complementary information from diffusion tensors and 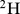 spectra imaged at sub-millimeter spatial resolution. On the time-scale of days and weeks, we follow the breakdown of MLVs after the cessation of shear and the formation of a uniformly oriented lamellar phase after a temperature quench, thereby obtaining structural information at an unprecedented level of detail. In addition to the time-dependence of the bulk phase composition during a phase transition, which is often analyzed with the Johnson-Mehl-Avrami-Kolmogorov model [45], [46], our approach also allows for identification of the nucleation sites and the three-dimensional growth pattern of the new phase.

## Methods

### Theoretical considerations

In this section we first briefly review the theoretical basis for 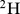 NMR spectroscopy and diffusion tensor imaging (DTI), and subsequently show schematic results that can be expected for typical micrometer-scale morphologies of lamellar phases.

#### ^2^H NMR

The 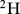 nucleus has a spin quantum number 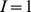 and an electric quadrupole moment, resulting in an NMR spectrum dominated by the interactions between the quadrupole moment and electric field gradients. For 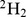
O in an isotropic liquid, the quadrupolar interaction is averaged to zero by molecular motion and the 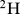 spectrum consists of a single sharp peak. Conversely, the preferential molecular orientation in an anisotropic liquid leads to a spectrum consisting of a doublet with splitting 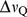 given by [47], [48]

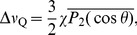
(1)where 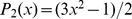 is the second Legendre polynomial, 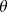 is the angle between the magnetic field and the O-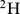 bond vector, and 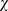 is the quadrupole coupling constant given by
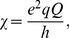
(2)where *e* is the unit charge, 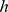 is the Planck constant, 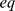 is the electric field gradient along the O-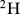 bond axis, and 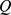 is the nuclear quadrupole moment. The value of 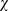 is 254 kHz for water at 25°C [49]. The overline in Eq. 1 indicates an average over fluctuations of 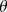 that occur much faster than the inverse strength of the interaction 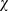. In anisotropic liquid crystals, the molecular motion is symmetric with respect to the phase director inclined at a polar angle 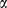 from the magnetic field. Assuming that the molecules remain in a domain with uniform value of 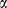 during the 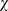 time scale, then Eq. 1 can be expressed as [50]

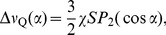
(3)where the order parameter 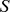 is given by
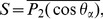
(4)and 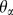 is the angle between the bond axis and the director. For water in typical surfactant lamellar phases, Eq. 4 evaluates to approximately 0.01, leading to observed quadrupolar splittings on the order of 1 kHz.

Lyotropic liquid crystals usually consist of an ensemble of randomly oriented anisotropic domains. The resulting powder-pattern 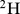 spectrum 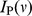 can be written as
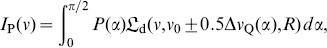
(5)where 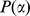 is the probability density of angles 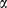, normalized in the interval 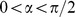, and 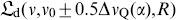 is a Lorentzian doublet lineshape function with peaks of width 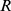 centered at the frequencies 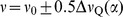. A random distribution of domain orientations in three dimensions corresponds to



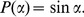
(6)The effect of preferential alignment in the magnetic field can be approximated by
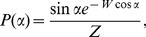
(7)where 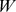 is a weighting parameter, corresponding to the degree of alignment, and 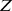 is a normalization factor assuring that 
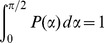
. The 3D powder case in Eq. 6 is recovered when 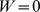. Complete alignment at 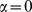 or 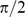 is obtained as 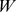 approaches 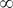 or 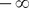, respectively.

Translational diffusion along the curved water layers in multi-lamellar vesicles (MLVs) may result in molecular reorientation on the millisecond time-scale of the rotationally averaged quadrupolar coupling, giving rise to nuclear relaxation and line broadening. As a consequence, the 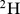 spectrum features a singlet with a linewidth that is proportional to the square of the MLV radius [24], [44]. The lineshape can in this case be approximated by
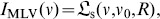
(8)where 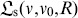 is a Lorentzian singlet with width 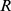 and centered at 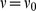.

For the purpose of visualizing 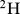 MRI data, it is useful to extract scalar parameters that describe the experimentally determined spectrum 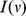. Here, we use the peak area 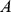, the first moment 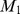, and the second moment 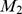:
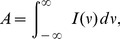
(9)

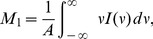
(10) and 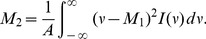
(11)


#### Diffusion tensor imaging (DTI)

Translational diffusion in a homogeneous and anisotropic medium can be described with the second rank diffusion tensor 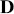, which is determined by its eigenvalues 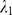, 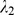, and 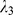 (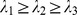) as well as the orientation of its eigenvectors with respect to the laboratory coordinate system 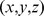. The transformation from the principal axis system (PAS) of the diagonalized diffusion tensor 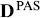 to the lab frame is brought about by
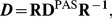
(12)where 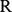 is a rotation matrix given by the three Euler angles.

In the pulsed-gradient spin-echo (PGSE) pulse sequence, the NMR signal is encoded for diffusion using two magnetic field gradient pulses of duration 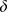, amplitude 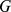, direction 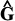, and time lapse between the leading edges 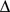. The detected signal intensity 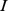 is expressed as
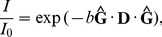
(13)where 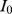 is the signal intensity at 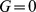 and the diffusion weighting variable 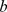 is given by
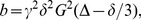
(14)where 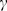 is the magnetogyric ratio. The scalar products in Eq. 13 can be explicitly written as



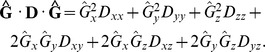
Experimentally, the diffusion tensor 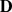 is estimated by analyzing the diffusional signal decay recorded for a series of 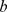-values and gradient directions 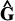 [51]–[53].

There are many ways to visualize the diffusion tensors, e.g., by plotting arrays of diffusion ellipsoids [34] or superquadrics [51], [54]. Alternatively, one can extract rotationally invariant indices such as the mean diffusivity (MD)
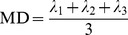
(16)and the fractional anisotropy (FA) [55], [56]

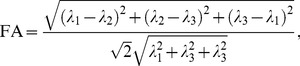
(17)as well as the linear (CL) and planar (CP) measures [57]

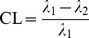
(18) and 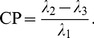
(19)


#### 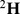 NMR, DTI, and lamellar phase morphology

**Figure 1:**
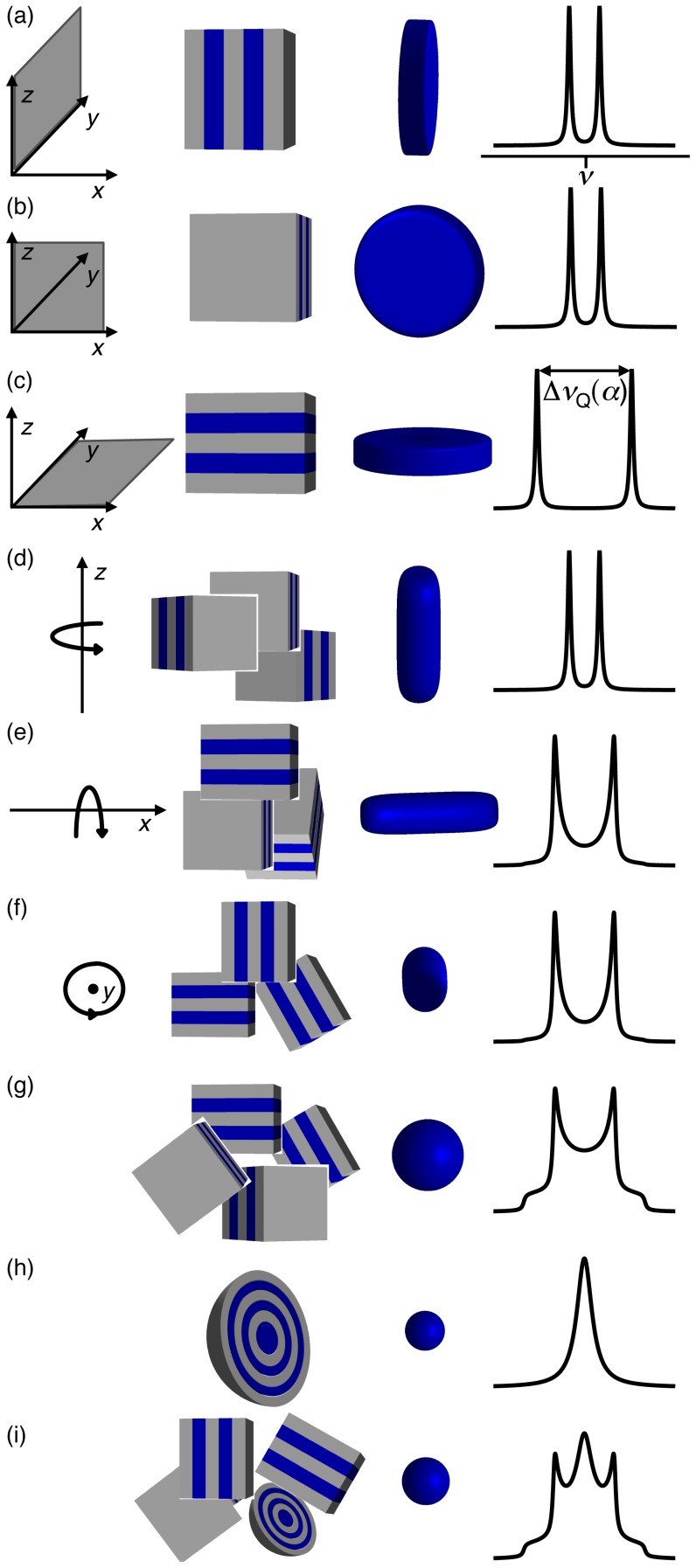
Schematic lamellar phase morphologies with corresponding diffusion tensors and 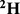 spectra. Column 1: Lab frame coordinate system indicating the direction of domain alignment. The main magnetic field 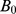 defines the direction of the 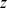-axis. Column 2: Schematic lamellar phase domains with water and surfactant shown in blue and gray, respectively. Column 3: Diffusion tensors displayed as superquadrics [54]. Column 4: 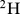 spectra with quadrupolar splitting 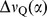, see Eq. 3. Rows (a), (b), and (c): Uniform orientation with non-restricted water diffusion in the 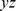, 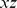, and 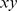-planes, respectively. Rows (d), (e), and (f): Domains oriented randomly along the curved arrow with main symmetry axis along 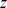, 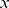, and 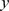-axes, respectively. Row (g): Random orientations in three dimensions. Row (h): Multi-lamellar vesicles (MLVs). Row (i): Mixture of MLVs and randomly oriented domains.

Surfactant/water lamellar phases can have a range of different morphologies on length scales above micrometers, e.g., uniformly or randomly oriented microdomains and multi-lamellar vesicles (MLVs). As illustrated with the schematic NMR data in Fig. 1, neither diffusion tensors nor ^2^H spectra are by themselves sufficient to unambiguously determine the microstructure. Still, all the different cases can be distinguished by combining the information from the two NMR modalities.

For a lamellar phase oriented with the director along 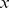 or 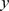, shown in Figs. 1 (a) and (b), the 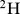 spectrum features a doublet split by 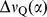. Noteworthily, there is no difference in the degree of splitting for single domain lamellar phases aligned with the magnetic field but rotated in the plane perpendicular to the magnetic field since the angle 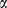 remains constant. Conversely, the orientation of the diffusion tensors directly mirror the orientation of the lamellae. Assuming 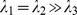, the values of the rotationally invariant diffusion tensor indices are given by 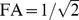, 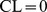, and 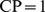 for all cases (a)-(c), while the lab-frame diffusion tensor elements 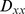, 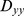, and 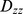 differ. The case with the lamellar director along 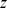 displays twice as large quadrupolar splitting as the 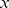 and 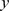 cases on account of the factor 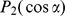 in the expression for 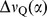 in Eq. 3.

A spread of domain orientations most often results in ^2^H powder line shapes [58] as illustrated in Figs. 1 (e)-(g), the only exception being if the different domains happen to have same angle 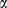 with respect to the magnetic field as shown in Fig. 1 (d). This seemingly unlikely case nevertheless appears quite often when the domains are in the presence of an aligning magnetic field. The diffusion tensors have cylindrical shapes for all the two-dimensional powders in Figs. 1 (d)-(f) despite the fact that the underlying compartment geometry is planar. The diffusion tensor indices evaluate to 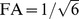, 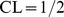, and 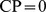.

The three-dimensional powder in Fig. 1 (g) results in a ^2^H powder line shape with characteristic “horns” and “shoulders”, while the diffusion tensor shows a spherical symmetry with 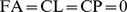. Diffusion along the curved water layers in the MLVs results in a 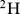 spectrum with a broad singlet rather than a doublet [24], see Fig. 1 (h). Isotropic diffusion tensors are obtained for both MLVs and 3D powders, the latter giving higher values of the mean diffusivity.

When several morphologies are present in the sample, the observed ^2^H spectrum is a superposition of the line shapes from all constituents. An example with coexistence between the 3D powder and MLVs is shown in Fig. 1 (i).

### Experimental

#### Sample preparation

The nonionic surfactant triethylene glycol monodecyl ether, C_10_E_3_, (Nikko Chemical Co., Tokyo, Japan) was mixed with deuterated water (Sigma Aldrich, Steinheim, Germany), yielding a final concentration of 40% (w/w) C_10_E_3_ and a molar ratio ^2^H_2_O/C_10_E_3_ of 24. All chemicals were used without further purification. The concentration of residual protons in the ^2^H_2_O is sufficient for performing ^1^H NMR experiments with adequate signal-to-noise ratio. The mixture was equilibrated overnight before being placed in a rheometer (PaarPhysica UDS 200, Hertford, United Kingdom, MK22/M; 1° cone angle). MLVs were formed at 25°C by applying 50 s^−1^ shear until reaching a viscosity plateau after 30–60 min.

After transferring the sample with the help of a syringe into a 5 mm outer diameter NMR tube, the evolution with time was followed by continuous NMR experiments for two weeks. After this set of NMR experiments, the sample was heated to 67°C into a two-phase region with reverse micelles (top) and essentially pure water (bottom) [14]. The temperature was rapidly lowered to 25°C and a second set of NMR experiments was performed for one week. Subsequently, the sample was removed from the magnetic field of the NMR equipment and equilibrated for an additional month at 25°C before a final set of NMR experiments. The combination of the temperature cycle and the presence of a magnetic field results in a uniformly oriented lamellar phase [6].

#### NMR experiments

NMR experiments were carried out on a Bruker AVII-500 spectrometer operating at ^1^H and 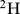 resonance frequencies of 500.13 and 76.77 MHz, respectively. The spectrometer was equipped with a 11.74 T standard bore magnet and a MIC-5 probe fitted with a 5 mm ^1^H/^2^H RF insert, allowing for simultaneous ^1^H and ^2^H studies. All NMR measurements were performed at 25°C.

For the 3D imaging experiments, great care was taken to get exactly the same field of view (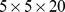 mm with imaging matrix size 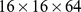 points) and spatial resolution (isotropic voxels with size 310* µm*) for both ^1^H and ^2^H experiments. One set of NMR experiments lasted 16 h. Gaussian smoothing with 300* µm* was applied to the spatial dimensions and an exponential weighting function with 10 Hz to the ^2^H spectral dimension. All data processing was performed in Matlab (MathWorks Inc., Massachusetts, USA) using in-house developed code, some of which derives from other sources [59], [60].

**Figure 2:**
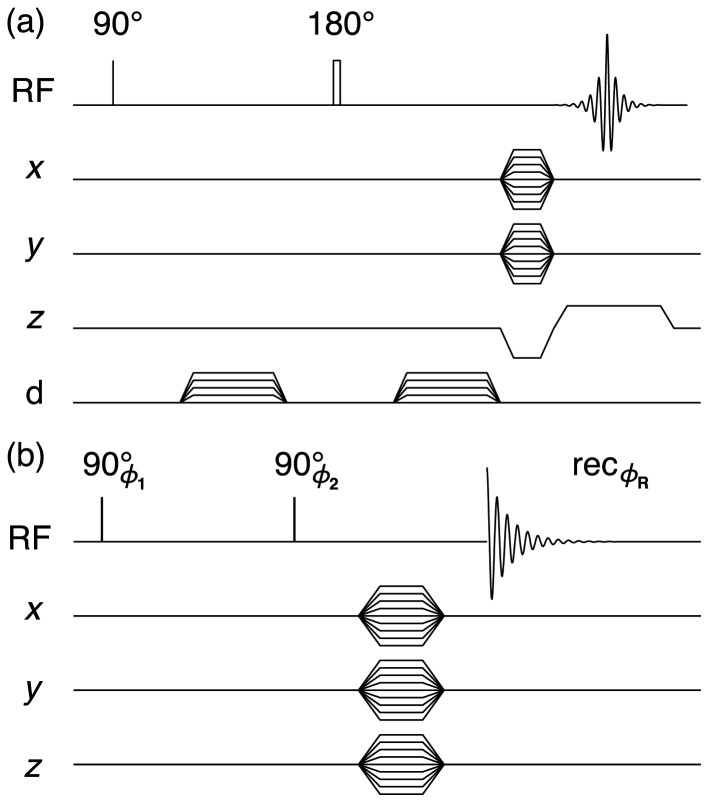
Timing diagrams of the NMR pulse sequences. (a) Diffusion tensor imaging (DTI) pulse sequence based on a ^1^H spin echo. Spatial resolution is provided by two phase-encoding gradients in the 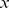 and 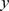-directions and frequency-encoding gradient in the 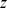-direction, while the diffusion gradients 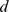 encode the images for translational motion. The phase and diffusion gradients are incremented independently, yielding a 4D experiment with three spatial and one diffusion dimension. The phase of the 180° RF pulse is cycled in two steps 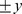. (b) 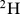 spectroscopic imaging sequence using a quadrupolar echo and three independently incremented phase-encoding gradients (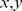 and 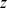-direction). The sequence yields a 4D data set with three spatial and one spectral dimension. The RF and receiver (rec) phases are cycled in 4 steps according to 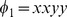, 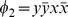, and 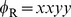.

The DTI pulse sequence in Fig. 2 (a) was used to measure the diffusion tensors with spatial resolution in three dimensions. The signal was acquired at an echo time of 31 ms. Diffusion gradients, with duration 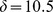 ms, separation between leading edges 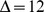 ms, and amplitudes 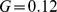 and 0.87 Tm^−1^, were applied in seven gradient directions: (1,0,0), (0,1,0), (0,0,1), (1,1,0), (1,0,1), (0,1,1), and (1,1,1). Combined with two measurements at 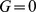, the two gradient amplitude increments and seven directions give a total of 16 gradient combinations in the diffusion dimension. Signal averaging over 2 scans and a recycle delay of 2 s result in an experimental time of 4 h and 40 min.

After Fourier transformation in the spatial dimensions, the diffusion tensors were estimated voxel-wise by non-linear least squares fitting of Eq. 13 with Eq. 12 to the experimental signal intensities using the initial signal intensity 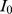, the eigenvalues 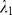, 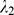, and 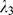, as well as the three Euler angles as adjustable parameters. For display purposes, the diffusion tensors were represented as superquadrics [54] and the DTI indices MD, FA, CP, and CL were calculated using Eqs. 16–19.

The longitudinal and transverse relaxation times 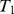 and 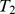 are about 1 s and 30 ms, respectively, for the residual water protons in the C_10_E_3_/^2^H_2_O mixture, giving some 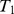
- and 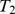-weighting of the signal at the herein used recycle delay and echo time. The relaxation weighting affects the value of 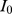 in Eq. 13, but should not alter the estimated diffusion tensors.

The quadrupolar echo pulse sequence in Fig. 2 (b) was used to record ^2^H spectra with spatial resolution in three dimensions. Signal acquisition was initiated at the top of the quadrupolar echo occuring 4 ms after the initial 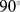 pulse. The signal was collected for 0.41 s with a spectral width of 2500 Hz and 2048 time domain points. With 0.2 s recycle delay and accumulation of 4 scans, the experimental time was 11 h and 22 min.

Fourier transformation along all four acquisition dimensions generated one ^2^H spectrum per voxel. The individual ^2^H spectra were processed with automatic phase and baseline correction. On account of our acquisition and processing protocol, including phase correction rather than the more common magnitude calculation, the voxel-resolved ^2^H spectra have a quality that is comparable to what can be obtained with conventional ^2^H spectroscopy without spatial resolution. The ^2^H spectrum indices 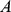, 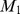, and 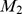 were calculated using Eqs. 9–11. The fractional population of water in the MLVs, 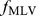, was estimated from the ^2^H spectra 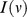 by non-linear least squares fitting of
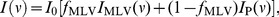
(20)where the spectrum from the MLVs, 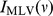, and the powder pattern 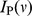 are given by Eqs. 8 and 5, respectively. In the evaluation of Eq. 5, the probability distribution of domain orientations 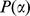 as expressed in Eq. 7 was used. The fitting was performed both with and without the constraint 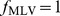. If there was no significant improvement of the fit quality by allowing 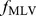 to vary, then 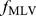 was set to unity.

While the values of 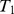 and 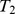 are approximately 0.5 s for pure 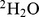, they could be considerably smaller and differ between the various lamellar morphologies, possibly leading to systematic errors in the estimated values of 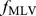. The relaxation weighting should be taken into account if the value of 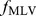 is in itself the parameter of interest, but it is of minor importance for the current study where we infer the rate of MLV breakdown from the change of 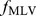 as a function of time.

## Results and Discussion

### Reference lamellar morphologies

**Figure 3:**
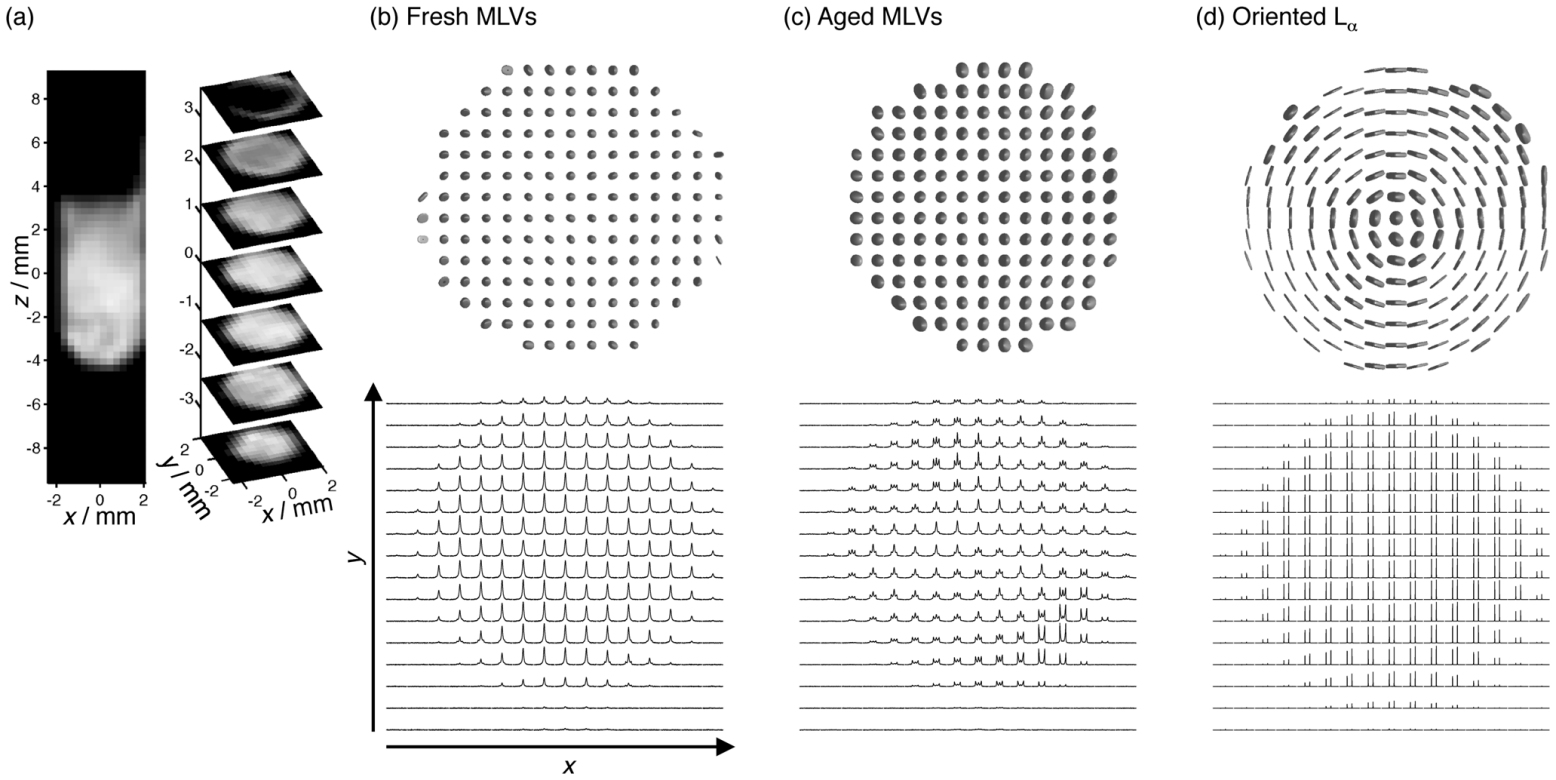
Representative DTI and 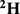 data for C_10_E_3_/water lamellar phases. (a) Selected 2D slices from a conventional 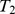-weighted ^1^H 3D image for a freshly prepared sample of multi-lamellar vesicles (MLVs). (b), (c), and (d): 2D arrays of diffusion tensors (top) and 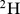 spectra (bottom) for fresh MLVs, 12 days aged MLVs, and an oriented lamellar phase L_α_ obtained by 36 days equilibration after a temperature quench. The 2D arrays show the 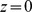 slices extracted from the full data sets with spatial resolution in three dimensions. Diffusion tensors are shown only for voxels with ^1^H signal intensity significantly above the noise level.

Fig. 3 shows DTI and 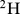 data for three representative C_10_E_3_/water lamellar phases with identical bulk chemical composition and temperature, but different morphology on length scales above micrometers because of sample history. The overall shape of the sample is visible in the standard ^1^H image in (a), which also shows the 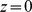 slice that is investigated in more detail in panels (b)-(d). Information about the sub-voxel morphology can be obtained by comparing the diffusion tensors and ^2^H spectra with the schematic data in Fig. 1.

The experimental data for the freshly prepared MLV sample agree well with the isotropic diffusion tensor and the broad 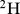 singlet shown for case (h) in Fig. 1. Visual inspection of the 2D arrays of diffusion tensors and 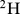 spectra in Fig. 1 (b) verify that the sample is nearly homogeneous. A few diffusion tensors with markedly flat shape are visible at the left-most voxels, possibly indicating the presence of a uniformly oriented lamellar phase, but more likely resulting from a failure of the DTI fitting process on account of the rather low signal intensity in these voxels. Still, careful inspection of the 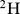 spectra reveals low-amplitude doublets in the immediate vicinity of the container walls, indicating that the MLVs have started to transform into other morphologies. When using standard 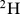 spectroscopy without spatial resolution, there is no trace of other morphologies than the MLVs (data not shown).

The transformation of the MLVs is apparent in the data for the aged MLV sample in panel Fig. 3 (c). The 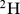 spectra show the presence of voxels with almost pure MLVs (single broad peak), as well as some voxels with seemingly uniformly oriented lamellar phase (doublet without “shoulders”). Voxel-by-voxel comparison with the diffusion tensors shows that the domain orientation is not completely uniform even in the voxels with the sharpest 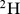 doublets. As a reference for this observation, we can use the data for the highly oriented lamellar phase in panel (d). All voxels show 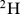 spectra with doublets having just a small fraction of the linewidth of the sharpest doublets in panel (c). Analogously, the diffusion tensors are flat, with values of the planar index CP approaching unity. Comparison to the schematic data in Fig. 1 shows that the lamellae are uniformly oriented within each voxel, and that the directors lie in the 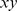-plane, pointing radially with respect to the axis of the sample tube. The diffusion tensors in the very center of the tube are cylindrical rather than planar, indicating that these voxels contain domains with directors spread out in the 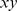-plane, corresponding to case (d) in Fig. 1.

**Figure 4:**
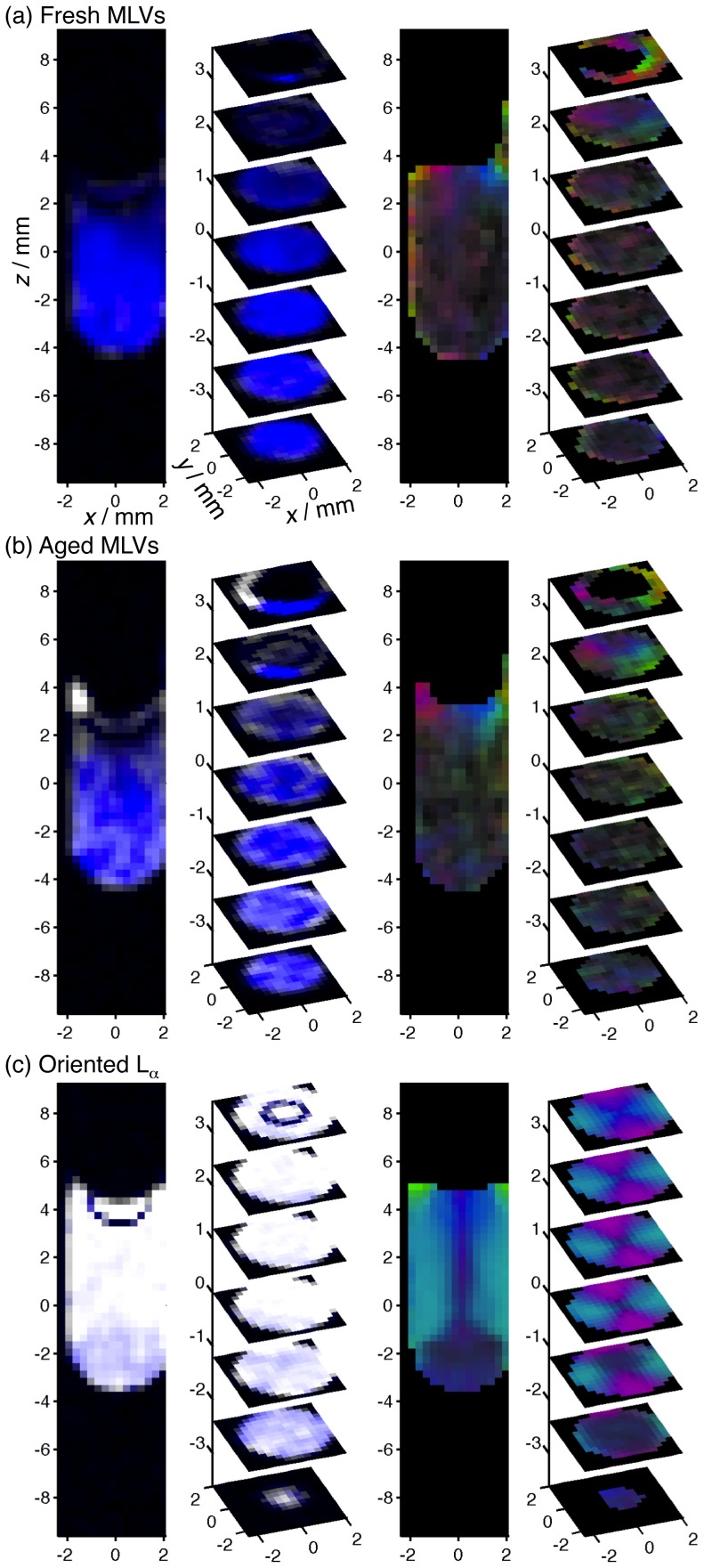
Color-coded 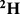 NMR and DTI data with spatial resolution in 3D. ^2^H NMR (left) and DTI (right) for C_10_E_3_/water lamellar phases with different superstructures: (a) fresh MLVs, (b) MLVs aged 12 days, (c) and oriented L_α_ equilibrated 36 days after a temperature quench. The voxels of the 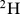 NMR data are color-coded with the 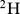 peak area 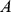 (brightness) and second moment 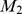 (narrow to broad peak: blue to white). The voxels of the DTI data are color-coded using RGB triplets calculated as FA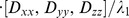.

In order to facilitate graphical display of the 3D data, some useful indices are extracted from the 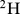 spectra and diffusion tensors, and shown as color-coded images in Fig. 4. The brightness of the 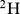 images is proportional to the total 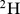 peak area 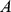 within each voxel, while the color-scale (from blue to white) corresponds to the 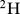 peak width expressed as the second moment of the linewidth 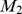. Intensely blue voxels thus show the presence of MLVs, while white voxels signify oriented lamellar phase. The black band below the meniscus is caused by the difference in magnetic susceptibility between the sample and the surrounding air, leading to inhomogeneity in the magnetic field and signal loss that cannot be refocused with the quadrupolar echo sequence. The DTI data is color-coded using RGB triplets calculated as [R, G, B]  =  FA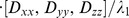. Consequently, the brightness is given by FA while the color gives visual cues for the shapes and orientations of the tensors. The purple and turquoise colors in panel (c) originate from diffusion tensors with 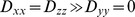 and 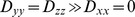, respectively, signifying diffusion tensors with planar shape, i.e. 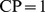, and uniformly oriented lamellar domains within each voxel.

The color-coded 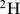 data in Fig. 4 is consistent with direct inspection of the 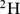 spectra in Fig. 3. The rather homogeneous blue and white colors in panels (a) and (c) correspond to homogeneous samples with MLVs and oriented L_α_, respectively. The pattern with blue tint in panel (b) shows the regions where the MLVs have partially transformed to other morphologies. The difference between the fresh and the aged MLV samples is less apparent in the DTI data, which however is quite powerful for showing the director orientations in the oriented L_α_ in panel (c). The director is located radially with respect to the tube axis, with the exception of the very bottom of the tube. In this particular region, which is also visible as a weak blue tint in the color-coded 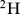 data, the 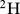 spectra feature sharp doublets, but with a few percent smaller splitting than in the rest of the sample. Tentatively, we attribute this observation to a small difference in chemical composition that remains even a month after the sample was quenched from the high-temperature phase-separated state with pure water in the bottom of the tube.

### Breakdown of multi-lamellar vesicles

In the presence of the externally applied shear field, the MLV phase is the thermodynamic equilibrium structure [61]. After turning off the shear, there is a thermodynamic tendency for a phase transition to the new equilibrium structure, i.e. a lamellar phase with flat rather than curved surfactant bilayers. Although the MLV phase is metastable for days, allowing for detailed investigation using techniques that are not compatible with the application of shear, the data in Figs. 3 and 4 show that the MLVs transform on the time scale of days, and that the transformation is spatially inhomogeneous.

**Figure 5:**
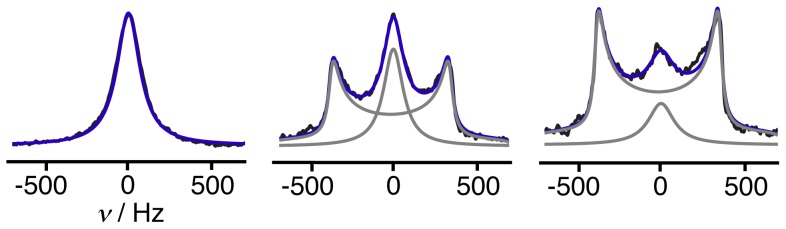
Estimation of the fraction of MLVs (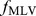) by spectral deconvolution of 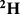 data. The observed spectrum is the sum of contributions from MLVs (singlet) and other lamellar morphologies (doublet). Experimental, fitted, and deconvoluted 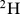 spectra are shown with black, blue, and gray lines, respectively. The deconvolution process yields the values 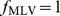, 0.32, and 0.14 from left to right.

In order to put these observation on a more quantitative basis, we use voxel-resolved estimates of the fraction of water residing in MLVs, 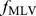, to segment the 3D images into regions that are dominated by either MLVs or other lamellar phase morphologies. The values of 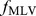 are obtained by deconvolution of the 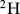 spectrum in each voxel, and Fig. 5 shows examples of the deconvolution process for three representative voxels extracted from the full 3D 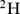 spectroscopic imaging data obtained on an aged MLV sample. The broad singlet at 0 Hz arises from the MLVs, whereas the powder-pattern doublet with maxima at 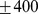 Hz originates from randomly orientated domains of lamellar phase.

**Figure 6:**
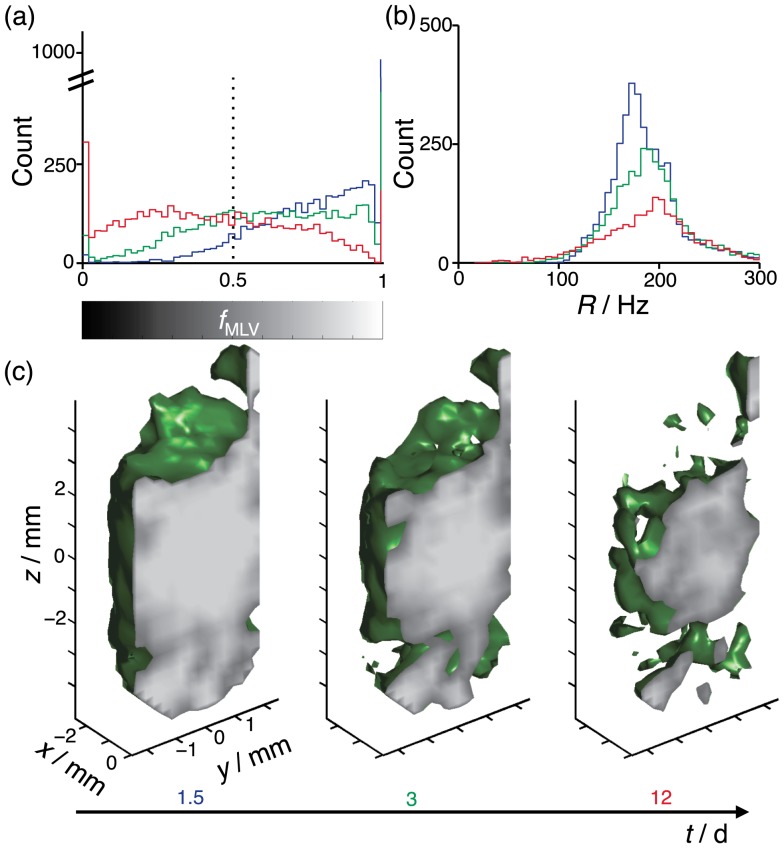
Time-resolved 3D mapping of the breakdown of MLVs to other lamellar morphologies. Spectral deconvolution of spatially resolved 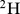 NMR spectra gives estimates of the fraction of MLVs 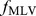 and peak width 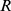 of the MLV singlet. Histograms of 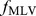 and 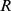 are shown in panels (a) and (b), respectively, for the times 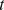 = 1.5 (blue), 3 (green) and 12 (red) days after MLV preparation. The dashed line in (a) indicates the value used for separating between voxels dominated by MLVs (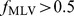) or other lamellar morphologies (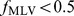). The counts in (b) are weighted by the values of 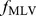. (c) 3D rendering of the contour 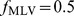 (green surface) at the times 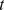 = 1.5, 3 and 12 days. The surface at 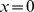 represents 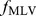 with gray scale given by the bar on the 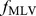-axis in panel (a).

The histograms in Fig. 6 represent the temporal change of 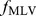 and width 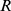 of the MLV singlet. The shift of the 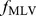-distribution towards smaller values reflects the breakdown of the MLVs with time. When constructing the 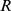-distributions, the contribution from each voxel was weighed by its value of 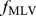, thus reducing the influence from voxels with low amplitude of the MLV peak. Consequently, the distributions are to a reasonable approximation weighted by the mass of water. With time, the main effect on the 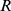-distribution is a decreasing amplitude on account of the decreasing values of 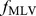, but also a shift of the maximum from 180 to 200 Hz, corresponding to a 5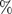 increase in size according to the proportionality between 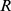 and the square of the MLV size [24], [44]. From these observations we conclude that the MLVs decrease in number while having a fairly constant size distribution.

The spatial pattern of the MLV breakdown is monitored by selecting voxels having 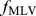 above 0.5. This threshold value is displayed as a sequence of 3D contour surfaces in Fig. 6 (c). We wish to point out that both the spatial and the temporal changes of 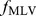 are always smooth, meaning that the surfaces in Fig. 6 (c) should be interpreted simply as a contour in the smoothly varying 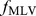 data rather than as a sharp interface between MLVs and other types of lamellar phases. The transition initially takes place close to the tube walls and then gradually progresses through the sample volume. Although the bulk of the sample has transformed, there are still isolated islands with pristine MLVs nearly two weeks after sample preparation.

**Figure 7:**
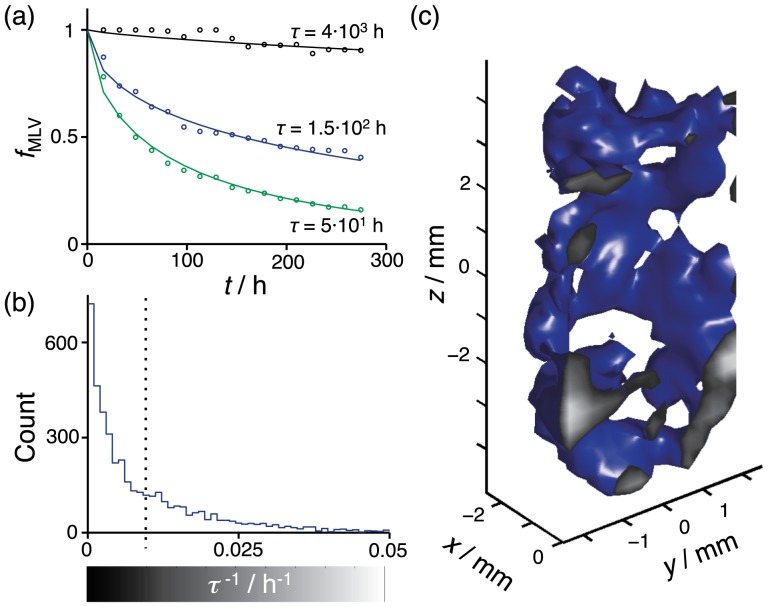
JMAK analysis of the transition from MLVs to a lamellar phase. (a) Fraction of MLVs 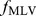 vs. time *t* for the entire sampe (blue) and two individual voxels (black and green). Fitting Eq. 21 (lines) to the experimental data (circles) gives estimates of the half-life time 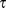. (b) Histogram of 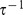 with the dashed line indicating the cut-off value used for separating between voxels with fast (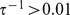 h^−1^) and slow (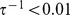 h^−1^) breakdown of the MLVs. (c) 3D rendering of the 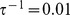 h^−1^ contour (blue surface). The surface at 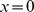 shows the values of 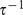 according to the gray-scale bar in (b).

The kinetics of phase transitions are often described by the Johnson-Mehl-Avrami-Kolmogorov (JMAK) model [45], [62]–[64], which has previously been applied to nonionic surfactant systems [46]. In the current context, we write the JMAK-model as
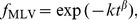
(21)where 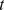 is the time, 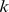 is the rate constant, and 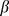 is the so-called Avrami constant. The half-life time 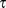 of the non-equilibrium phase is calculated by



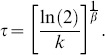
(22)Conventionally, the JMAK model is applied to the total phase composition in bulk samples. Each voxel of the 3D image has a volume of 30 nL and initially contains on the order of 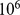 MLVs, and can thus be treated as an individual sample giving spatially resolved values of 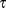. The JMAK model describes the experimental data well as shown for representative voxels in Fig. 7 (a). The voxels are chosen to illustrate the many orders of magnitude spread in the values of 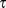, from 50 to at least 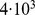 h. The upper limit of 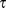 is difficult to determine since the experiment was terminated after 300 h.

Fig. 7 (b) shows a histogram of 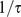, revealing a continuous distribution of breakdown rates. A 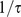 threshold value of 0.01 h^−1^, which splits the distribution into two halves with approximately equal areas, was chosen to discriminate between voxels with short- and long-lived MLVs. This threshold value is rendered as a 3D surface in Fig. 7 (c), showing that there is a tendency for voxels with short-lived MLVs to be located in the vicinity of the tube walls.

A phase transition involving a nucleation-and-growth mechanism with only a few separated nucleation sites would presumably lead to some voxels displaying an initial lag time, without changes in 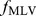, before the onset of a rapid phase transition as the equilibrium phase grows from the nucleation sites and eats its way into the rest of the sample. Voxels with such behavior could not be found, thus indicating that the MLVs transform with a rate that is given by the initial conditions within the voxel. Further discussions on the phase transition mechanism is beyond the scope of this paper, but we wish to emphasize that our experimental approach gives highly detailed data that could be used to test models for the mechanisms.

### Alignment of a lamellar phase

As a final example, we show the transition between randomly and uniformly oriented lamellar phases. The experiment was performed on a sample that was initially phase separated at 67°C into a reverse micellar phase (top) and pure water (bottom), both of which are isotropic. After a temperature quench to 25°C, anisotropic phases form throughout the sample after approximately 1 day. At this time, the combination of information from the 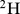 and DTI data shows that lamellar phases of various morphologies have formed throughout the sample despite the fact that there is still a gradient in chemical composition along the 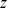-axis.

**Figure 8:**
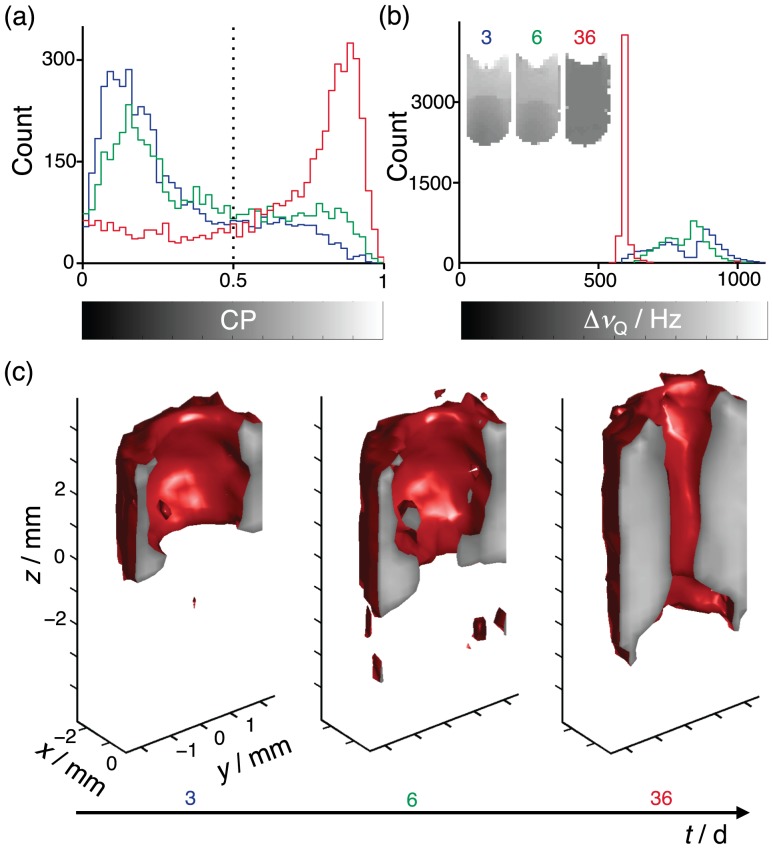
Time-resolved 3D mapping of the formation of a macroscopically oriented lamellar phase. Panels (a) and (b) show histograms of the planar diffusion anisotropy index CP and the ^2^H quadrupolar splitting 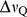 at the times 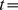 3 (blue), 6 (green), and 36 (red) days after a temperature quench from 67 to 25°C. The vertical dashed line in (a) indicates the value of CP for segmenting the 3D image into oriented (CP 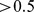) and non-oriented (CP 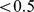) lamellar phase. Insets in (b) show 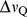 maps at 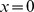, gray-scale coded according to the bar below the 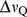-axis. (c) 3D rendering of the 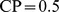 contour (red surface) for the times 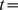 3, 6, and 36 days after the temperature quench. The surface at 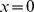 indicates the value of CP according to the gray-scale bar in (a).

Histograms of the planar index CP and the 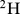 quadrupolar splitting 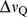 are displayed in Fig. 8 (a) and (b), respectively. Values of CP approaching unity indicate the presence of uniformly oriented lamellar phases, whereas 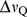 is proportional to surfactant-to-water ratio [41], [44]. The insets in (b) show 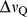 maps, revealing an initially heterogeneous sample composition that gradually becomes homogeneous. The CP distributions have significant amplitude over the entire range from 0 to 1, but also well-defined maxima at 0.1 and almost 1. The spatial distribution of the oriented lamellar phase is mapped by segmenting the 3D image using the threshold value CP 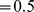. As opposed to the case with the MLVs discussed above, there is a distinct step in CP between voxels with uniformly and randomly oriented lamellar phases, resulting in a sharp and well-defined interface between the two morphologies.

The gradual formation of an oriented lamellar phase is illustrated by the sequence of 3D images in Fig. 8 (c). Initially, the oriented phase forms at the container walls in the top region of the sample where the surfactant concentration is highest. With time, the ordered phase grows downwards along the walls of the tube, and inwards towards the center of the tube. In the time period between 7 and 35 days after the temperature quench, the sample was stored far from the strong magnetic field of the NMR magnet. Still, the oriented phase kept on growing, finally filling the major part of the tube. From this observation we conclude that interaction with the glass walls of the tube is the main factor for the alignment of the C_10_E_3_/water lamellar phase.

## Conclusions

The experimental results presented in this paper show that the combination of data from 3D diffusion tensor and 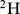 spectroscopic imaging yields a wealth of information on the morphology of liquid crystalline structures on a range of length scales. The combination of methods is necessary since they by themselves fail to distinguish between certain morphologies, e.g., MLVs and randomly oriented lamellar phases for DTI, as well as uniformly and 2D randomly oriented lamellar phases for 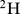. Image segmentation based on appropriately chosen scalar parameters and confirmed by voxel-by-voxel visual inspection of diffusion tensors and 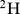 spectra allow for time-resolved 3D mapping of the spatial distribution of lamellar morphologies during phase transitions and subsequent equilibration. The combination of methods may prove to be useful in fundamental studies of phase transition mechanisms and for characterization of tailor-made metastable morphologies being produced by, e.g., temperature cycling or the application of external fields. The current implementation of the measurement protocol is limited to samples that remain stable on the time-scale of tens of hours, but we expect that the time resolution could be greatly improved by compressed sensing approaches with sparse sampling of the acquisition dimensions [65].
