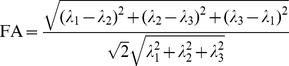# Correction: Multi-Scale Characterization of Lyotropic Liquid Crystals Using ^2^H and Diffusion MRI with Spatial Resolution in Three Dimensions

**DOI:** 10.1371/journal.pone.0109499

**Published:** 2014-09-26

**Authors:** The PLOS ONE Staff

In the Methods section, there is an error in equation 17 under “Diffusion tensor imaging (DTI).” The denominator is incorrect. Please view the complete, correct equation 17 here: